# Hormonal Influences on Pod–Seed Intercommunication during Pea Fruit Development

**DOI:** 10.3390/genes13010049

**Published:** 2021-12-24

**Authors:** Mark Bal, Lars Østergaard

**Affiliations:** John Innes Centre, Norwich NR4 7UH, UK; mark.bal@jic.ac.uk

**Keywords:** auxin, 4-chloroindole-3-acetic acid, trehalose-6-phosphate, starch, reproduction, development, pea, signalling

## Abstract

Angiosperms (from the Greek “angeion”—vessel, and “sperma”—seed) are defined by the presence of specialised tissue surrounding their developing seeds. This tissue is known as the ovary and once a flower has been fertilised, it gives rise to the fruit. Fruits serve various functions in relation to the seeds they contain: they often form tough physical barriers to prevent mechanical damage, they may form specialised structures that aid in dispersal, and they act as a site of nutrient and signal exchange between the parent plant and its offspring. The close coordination of fruit growth and seed development is essential to successful reproduction. Firstly, fertilisation of the ovules is required in most angiosperm species to initiate fruit growth. Secondly, it is crucial that seed dispersal facilitated by, e.g., fruit opening or ripening occurs only once the seeds have matured. These highly coordinated events suggest that seeds and fruits are in close communication throughout development and represent a classical problem of interorgan signalling and organismic resource allocation. Here, we review the contribution of studies on the edible, unicarpellate legume *Pisum sativum* to our understanding of seed and fruit growth coregulation, and propose areas of new research in this species which may yield important advances for both pulse agronomy and natural science.

## 1. Introduction

How multicellular organisms regulate the development of their reproductive structures, and how this process intersects with the development and growth of their offspring, are areas of active research in molecular biology. From the perspective of resource allocation, reproductive tissue development presents distinctive problems. The potential failure of fertilisation or embryo formation makes extensive investment (of nutrients, time, or water) in pre-fertilisation structures economically precarious. As a result, many organisms produce pre-fertilisation reproductive structures that are far smaller than the total size of the mature reproductive organ. As offspring viability is the determining factor in whether further investment in a reproductive organ is beneficial, organisms have developed complex systems for sensing the status of their offspring within their reproductive tissues. In flowering plants, failure of the offspring to develop may result in the spontaneous abortion of the entire reproductive organ, which prevents further investment into non-viable fruit. This can, in turn, free up resources for reallocation into other, viable developing reproductive structures, or into the survival of the parent plant as a whole.

Pea (*P. sativum*) is a crop plant species with an extensive history in genetics research [1], and a useful model for legume crops in general. Unlike the most regularly used model angiosperm, *Arabidopsis thaliana*, which produces bicarpellate (two-chambered) fruit [2] and many tiny seeds, pea produces monocarpellate (single-chambered) fruit (called pods) and a smaller number of comparatively much larger seeds [3]. Deciphering the seed/fruit molecular conversation that regulates pea reproductive development may lead to the discovery of not only the different signalling systems relative to *Arabidopsis*, but perhaps also to important advancements in legume agronomy in general, as pod shape varies between crop accessions [4]. Here, we review research on seed/pod intercommunication in pea, present outstanding questions, and provide avenues for future research.

## 2. Pod Elongation: 4-Chloroindole-3-acetic Acid as a Possible Seed-to-Pod Mobile Signal

### 2.1. Biosynthesis of 4-Chloroindole-3-acetic Acid

Phytohormones are a well-established means by which plants control and coordinate the development of their organs. Auxin (indole-3-acetic acid, IAA) is the most extensively studied phytohormone, and the primary mechanism of its perception is well-characterised (reviewed in [5]). In brief, auxin’s canonical role involves altering the expression of genes which are downstream of auxin-sensitive promoters containing auxin-responsive cis-regulatory elements (AuxREs). AuxREs are bound by transcriptional regulator proteins known as auxin response factors (ARFs), which, in the absence of auxin, are themselves bound by the transcriptional repressor proteins known as Aux/IAAs. Auxin acts by promoting interaction between Aux/IAAs and the transport inhibitor resistant1/auxin response F-box (TIR1/AFB) subunit of the SCF E3 ubiquitin ligase complex [6], hence promoting the degradation of Aux/IAA repressors. This, in turn, allows the now liberated ARFs to interact with proteins which alter gene expression, such as ATP-dependent chromatin remodelling proteins or histone-targeting lysine deacetylases (HDACs) [7]. Only class A ARFs upregulate the expression of their bound genes, however, as B and C class ARFs lack the glutamine-rich middle region necessary for activatory functions [8]. Other non-canonical modes of auxin response have also been described [9]. Auxin is a highly versatile hormone affecting virtually all aspects of plant development, including the formation of fruits [10,11]. It is therefore possible that auxin is also involved in mediating pod/seed intercommunication.

**Figure 1:**
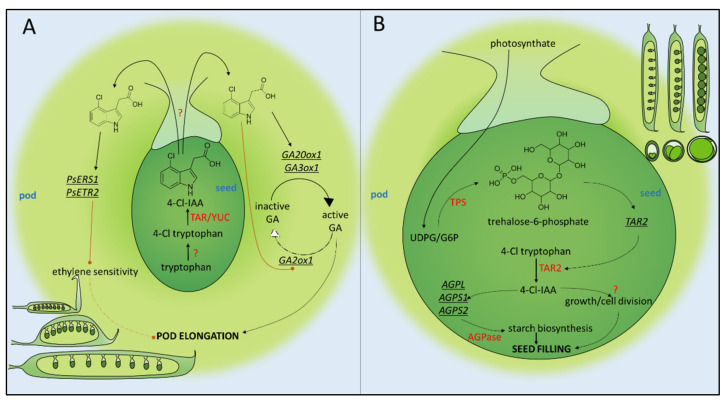
Schematic representation of pod elongation (**A**) and seed filling (**B**) stages of pea reproductive development. 4-Cl-IAA, 4-chloroindole-3-acetic acid; TAR, tryptophan aminotransferase related; YUC, yucca; GA, gibberellic acid; GAox, gibberellic acid oxidase; ERS1, ethylene response sensor 1; ETR1, ethylene receptor 1; UDPG, uridine diphosphate glucose; G6P, glucose-6-phosphate; TPS, trehalose phosphate synthase; AGPase, adenosine diphosphate glucose pyrophosphorylase.

Peas (along with other species in the Fabeae and Trifolieae clades of Fabaceae [12]) produce a chlorinated variant of auxin by an unknown mechanism [13] (Figure 1A). Bearing a chlorine atom at the 4′ position of its indole ring, 4-chloroindole-3-acetic acid (4-Cl-IAA) is enriched in fruit and seed tissues, relative to vegetative tissues, but its biological function is yet to be understood [14]. IAA levels in developing seeds are initially higher than 4-Cl-IAA levels, but this reverses 7–12 days after anthesis (anthesis is defined as the full reflex of petals) when 4-Cl-IAA begins to predominate [15]. The exact physiological significance of 4-Cl-IAA is unclear, but exogenously applied 4-Cl-IAA was capable of promoting pericarp elongation in deseeded pods, whilst non-chlorinated IAA was unable to do so [16]. In this context, 4-Cl-IAA has been suggested to fulfil the role that intact, fertilised seeds ordinarily play in stimulating fruit growth, and endogenous 4-Cl-IAA is therefore suspected to be a seed-to-fruit mobile signal. To date, no mutant lacking the ability to produce 4-Cl-IAA has been identified in pea, which makes the relative contribution of 4-Cl-IAA to fruit growth and development (as opposed to the contribution of non-chlorinated IAA) difficult to appreciate. Moreover, the significance of the evolution of this hormone ~25 million years ago [12] within a pair of Papilionid clades (Trifolieae and Fabeae) and its absence from the sister taxon Cicereae, and all other earlier diverging plant species, is unclear.

**Figure 2:**
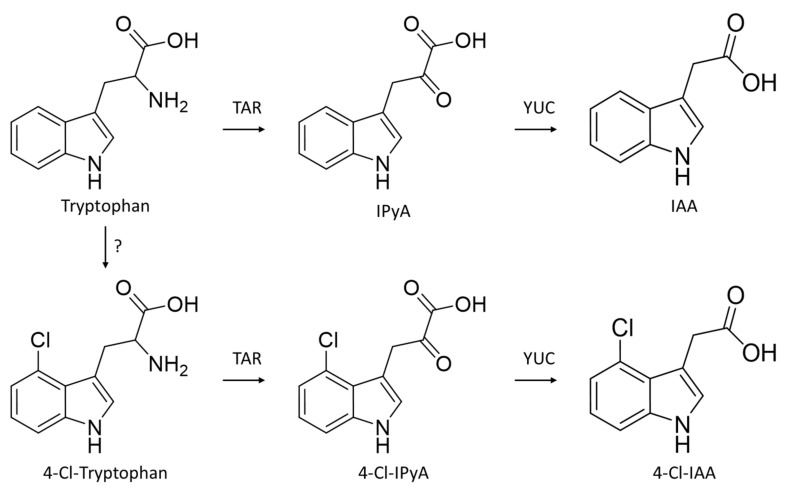
Biosynthesis of auxin and 4′ position chlorinated auxin in pea. Enzyme responsible for halogenation of tryptophan is unknown. IPyA, indole pyruvic acid; 4-Cl-IPyA, 4-chlorinated indole pyruvic acid; IAA, indoleacetic acid; 4-Cl-IAA, 4-chloroindole acetic acid. Schema based on results from [15].

Generating mutants lacking 4-Cl-IAA as part of a reverse-genetic approach, such as via Targeting Induced Local Lesions in Genomes (TILLING [17,18]) or Clustered Regularly Interspaced Short Palindromic Repeats and CRISPR-associated protein 9 (CRISPR-Cas9 [19]), is not currently possible due to the partially unresolved biosynthetic pathway of this rare phytohormone. As the pea mutant lacking the auxin biosynthesis gene *TRYPTOPHAN AMINOTRANSFERASE RELATED2* (*TAR2*) had reduced levels of 4-Cl-IAA [15], and because chlorinated tryptophan was detected in pea tissues [15], it is currently thought that 4-Cl-IAA is produced from 4-Cl tryptophan, which is itself generated from tryptophan or a tryptophan precursor by an unknown halogenating enzyme. Both tryptophan and 4-chlorotryptophan are then converted into indole-3-pyruvic acid (IPyA) and 4-chloroindole-3-pyruvic acid, respectively, by TAR enzymes. These intermediates are then, in turn, converted into IAA and 4-Cl-IAA by YUCCA (YUC) enzymes (Figure 1). The committal step in the production of 4-Cl-IAA, therefore, appears to occur at (or above) the amino acid level, as a result of halogenase activity, and before each tryptophan species is then converted into auxin (Figure 2).

Halogenating enzymes, while relatively common in bacteria, are rare in plants [20], and discovering the identity of the enzyme responsible for 4-Cl tryptophan in pea is a critical next step in our understanding of 4-Cl-IAA’s biological function. Currently, the only enzymes known to be capable of halogenating indolic molecules, such as tryptophan, come from bacteria and fungi, and are known as flavin-dependent halogenases, or FDHs. All flavin-dependent tryptophan halogenases share two absolutely conserved motifs that are required for their catalytic activity: GxGxxG and WxWxIP [21]. Whilst several pea proteins contain one of these motifs, we were not able to identify any pea protein sequences containing both of these motifs in the publicly available database [22]. Additionally, our BLASTs of the public pea genome [22] indicated a total absence of genes related to bacterial-like *FDHs*. We speculate that the *Pisum* halogenase is not homologous to bacterial *FDHs* or has diverged significantly in sequence. An alternative hypothesis is that 4-chlorotryptophan is produced by bacteria resident in the *Pisum* carposphere (fruit), as at least one other halogenated molecule was discovered to be a phyllosphere product in another plant species [23]. Even among bacteria, however, an FDH enzyme shown to be capable of producing chlorinated tryptophan at position 4 (4-Cl-Tryp) has yet to be identified in nature.

The absence of *FDHs* from the pea genome has led to the hypothesis that the enzyme responsible for the production of 4-chlorotryptophan may be a haloperoxidase enzyme [12], as peroxidases are widespread in plant genomes. Haloperoxidases operate by using hydrogen peroxide as an oxidant to convert halide ions into hypohalides, which can then react with an organic substrate [24]. There are no haloperoxidases known to be capable of halogenating tryptophan, however, and the low substrate specificity in this family makes them unlikely to be capable of the required selectivity to produce 4-chlorotryptophan in pea [21]. Auxins that are chlorinated at other positions (5-, 6-, or 7-Cl IAA) do not naturally occur in pea, and when synthesised and exogenously applied to deseeded fruit, have been found to be ineffective in rescuing pod growth [25].

### 2.2. Activity of 4-Chloroindole-3-acetic Acid in Promoting Pod Elongation

Reinecke and colleagues [16] developed a split pericarp assay in which the dorsal suture of a young pea pod was surgically opened, allowing access to its developing seeds and to the pod’s interior surfaces. The retention of seeds in the pod allows for the growth and development of the fruit to continue, though the researchers noted that dorsal cutting reduced pod length relative to intact pods [16,25]. Across multiple studies [16,25], they observed that the removal of the seeds 2–3 days after anthesis gradually halted pod growth and led to pod abscission. Exogenous applications of IAA to deseeded fruit did not restore pod growth or prevent abscission, and in fact, even resulted in a final pericarp length that was shorter (20 mm) than the deseeded pericarps with no hormone treatment (25 mm) [16]. Exogenous applications of 4-Cl-IAA, however, rescued deseeded pericarp growth in a concentration-dependent manner [25]. The daily application of as little as 1 µM 4-Cl-IAA was found to increase pericarp growth relative to the deseeded controls [25]. The ability of exogenous 4-Cl-IAA to rescue deseeded pericarps when exogenous IAA could not suggests that the cells of the pea fruit may be able to discriminate between these two auxin variants. This could be at the level of signalling, cell-to-cell transport, and/or auxin conjugation.

Pioneering experiments carried out by Reinecke, Ozga, and colleagues [16] revealed that substituting the chlorine atom in position 4 of 4-Cl-IAA with other functional groups could alter, but not always abrogate, the ability of the hormone to rescue deseeded pod growth. Of the 4-substituted auxins, 4-methyl IAA had the second strongest growth-promoting activity after 4-Cl-IAA, while the alternative halogenated auxin, 4-fluoro IAA (4-F-IAA) showed no ability to rescue pericarp growth [16]. These surprising results showed that the electronegativity of the functional group in position 4 had little effect on the molecule’s ability to rescue the growth of the deseeded pericarps. The researchers concluded that the structural recognition of 4-Cl-IAA is at least not entirely based on local charge. Instead, the properties of functional group size and lipophilicity were found to be the strongest predictors of the ability of a 4-substituted auxin to rescue pod growth. In nature, it is possible that the presence of the chlorine atom alters the equilibrium conformation of the ethanoic acid group of the molecule, or alters overall lipophilicity, and that this in turn alters its Kd to a sensing protein relative to non-chlorinated IAA.

A classical system for measuring a plant’s response to exogenous auxin comes from the *Arabidopsis* seedling root inhibition assay, in which plants are grown on medium containing auxin which inhibits seedling root elongation. *Arabidopsis* root growth responds to several auxinic compounds in addition to IAA, such as the synthetic auxins NAA and 2,4-D. Testing the effect of 4-Cl-IAA on *Arabidopsis* root elongation revealed it to be a more potent inhibitor than IAA [26]. Additional evidence for a potency disparity was indicated by using the synthetic auxin-signalling reporter *DR5::GUS* [27]. Using the *DR5::GUS* reporter in pea revealed increased auxin signalling in pea pods treated with 4-Cl-IAA compared to equimolar IAA. These results suggest that 4-Cl-IAA at least partially functions through the canonical auxin signalling pathway mediated by the TIR1/AFB auxin receptors. Indeed, functional *TIR1/AFB* genes were identified in pea that were able to rescue the *Arabidopsis tir1-10 afb2-3* double mutant [23]. The significance of the differential effect of 4-Cl-IAA and IAA on wild-type *Arabidopsis* root inhibition is unclear, as *Arabidopsis* does not naturally produce 4-Cl-IAA. This may indicate that some components of the auxin signalling system that are common to both legumes and Brassicaceae are sufficient for auxin discrimination. Studies on whether 4-Cl-IAA and IAA show differential effects when exogenously applied to very early diverging plants, such as *Marchantia*, may prove informative in further elucidating the common components of the differential perception mechanism. This may not be fully revealing, however, as there could conceivably be components of the *Pisum* auxin signalling, transport, or conjugation systems which further alter plant responses to each hormone. This would be consistent with the apparently qualitative differences in the responses of pea fruit to 4-Cl-IAA and IAA, as opposed to the quantitative differences observed in *Arabidopsis* root inhibition.

The abilities of 4-Cl-IAA and IAA to modify the transcript abundance of the three *Pisum* TIR/AFB homologs, *PsTIR1a*, *PsTIR1b*, and *PsAFB2*, were also explored. Deseeding fruit was associated with a threefold increase in the expression of *PsTIR1b*, which an exogenous application of 4-Cl-IAA, but not IAA, was able to attenuate [26]. Across timepoints and treatments, the transcript abundances of *PsTIR1a* and *PsAFB2* remained relatively constant. It is therefore possible that 4-Cl-IAA may operate *in planta* partly by the suppression of *TIR1b* expression, but why IAA is not capable of altering expression in the same way remains unclear.

### 2.3. Secondary Hormonal Regulation of Pod Elongation

Though the differential perception mechanism between 4-Cl-IAA and IAA remains to be fully elucidated, there is strong evidence that 4-Cl-IAA stimulates fruit growth by promoting gibberellic acid (GA) signalling. Gibberellins exist in a variety of active and inactive forms. The active forms (e.g., GA_1_ and GA_3_) bind to their receptor GID1 and promote the interaction of GID1 with DELLA proteins, which are negative regulators of plant growth. The GID1/GA/DELLA trimolecular complex can then interact with the F-box protein SLY1 to promote DELLA ubiquitination and degradation via the SCF E3 ubiquitin ligase complex [28]. GA_3_ was found to be capable of the independent rescue of growth in deseeded pericarps and exhibited a synergistic effect when applied in combination with 4-Cl-IAA [29]. 4-Cl-IAA was also shown to restore the expression of pro-bioactive GA biosynthesis enzymes *GA 20-oxidase1* (*GA20ox1*) [29] and *GA 3-oxidase1* (*GA3ox1*) [30] in deseeded pericarps and downregulated *GA2ox1* expression, which converts bioactive GA_1_ into inactive GA_8_ [31]. The net effect of exogenous 4-Cl-IAA seems to be to promote high equilibrium concentrations of bioactive GA when applied to deseeded pericarps, which may underlie its growth-promoting activity.

The combinatorial addition of GA_3_ and IAA to deseeded pericarps reduced pericarp growth relative to the GA_3_ exclusive treatment [32]. This unexpected result strongly indicates that IAA may negatively regulate pod growth. This negative effect of IAA/GA_3_ treatment was attenuated by additional treatment with silver thiosulfate (STS), an ethylene signalling inhibitor, which suggests that IAA inhibits GA_3_-stimulated pericarp growth by promoting ethylene signalling. This is supported by the observation that ethephon (an ethylene evolving agent) reduced the final fresh weight of deseeded pericarps relative to the deseeded controls [32]. Measurements of ethylene evolution under different hormonal treatments revealed that both IAA and 4-Cl-IAA stimulated the production of similar quantities of ethylene, but 4-Cl-IAA-induced growth was not abrogated by the addition of ethephon [32].

The differential effects of IAA and 4-Cl-IAA are thought to result from differences in ethylene-related gene expression elicited by the two auxins [33]. The ethylene response in plants has been extensively studied (reviewed in [34]). In the absence of ethylene, a suite of ethylene receptors (e.g., ETR1, ETR2, ERS1, ERS2, and EIN4) activate the kinase CTR1, which in turn phosphorylates EIN2. This prevents EIN2 from inhibiting the SCF E3 ubiquitin ligase-mediated degradation of transcription factors EIN3 and EIL1, which occurs via the F-box proteins, EBF1 and EBF2. When ethylene binds to its receptors, it prevents them from activating CTR1 and in turn prevents the phosphorylation of EIN2. This leads to the cleavage of EIN2′s C-terminal domain (EIN2-C), which inhibits the ubiquitination of EIN3/EIL1 by promoting the degradation of the mRNA encoding EBF1 and EBF2, thereby facilitating EIN3/EIL1-mediated gene expression. EIN2-C is also thought to translocate to the nucleus where it upregulates EIN3/EIL1 activity. 4-Cl-IAA, but not IAA, was found to significantly increase the transcript abundance of ethylene receptor genes *PsERS1* and *PsETR2* when exogenously applied to deseeded pericarps [33]. Conceivably, this could lead to an increase in the abundance of the ethylene receptors at the protein level, which in turn could increase the threshold level of ethylene required to overcome the constitutive activation of CTR1. Furthermore, 4-Cl-IAA treatment increased *PsEBF1* and *PsEBF2* transcript abundance, which may lead to an accelerated degradation of EIN3/EIL1. Hence, it is possible that 4-Cl-IAA partly acts by decreasing the sensitivity of young fruits to ethylene, and this explains why 4-Cl-IAA-stimulated growth is resistant to ethylene produced both from the application of 4-Cl-IAA itself and from exogenous ethephon. IAA appears to lack this ethylene desensitising activity relative to 4-Cl-IAA [33].

## 3. Seed Filling: Trehalose 6-Phosphate/Auxin Signalling

Trehalose-6-phosphate (T6P) is a phosphorylated disaccharide that is widespread in plants (reviewed in [35]). Canonically, T6P is an intermediate metabolite in the trehalose biosynthetic pathway, synthesised from glucose-6-phosphate (G6P) and UDP-glucose (UDPG) by trehalose phosphate synthase (TPS) (Figure 1B). T6P is then, in turn, de-phosphorylated by trehalose phosphate phosphatases (TPPs) to produce trehalose.

T6P is implicated in positively regulating starch biosynthesis by the post-translational redox activation of ADP-glucose pyrophosphorylase (AGPase). AGPase is a plastidic enzyme responsible for starch granule initiation, and is activated by the reduction of its autoinhibitory disulphide bridges [36]. Experiments involving feeding trehalose to potato and *Arabidopsis* leaf discs, the genetic manipulation of *Arabidopsis* trehalose metabolism, and experiments in which T6P was fed to isolated pea chloroplasts [37] have been shown to alter AGPase activation. T6P is also thought to be involved in development, including embryo growth [38], induction of flowering [39], growth bursts after periods of cold [40], and axillary bud activation [41]. Its status as a sugar, as well as its involvement in both metabolic and developmental processes, has led to the widespread paradigm that T6P is involved in coordinating plant growth in relation to carbon supply [42].

The *Arabidopsis* genome encodes only a single widely expressed TPS enzyme (AtTPS1), and when the gene encoding this enzyme was mutated, embryos failed to develop beyond the torpedo stage [38]. To better elucidate the role of T6P in seed development, Meitzel et al. [43] generated transgenic pea lines with an elevated embryonic expression of either *TPS* or *TPP*, by expression of heterologous *Escherichia coli* genes *otsA* and *otsB*, in conjunction with an embryo-specific promoter *USP* [44].

Meitzel et al. [43] found that in *proUSP:TPP*-expressing embryos, reduced embryonic T6P was associated with defects in the seed-filling stage of embryonic development, in which incoming carbohydrates are converted into starch and stored in the seed [43]. Embryos expressing heterologous *TPP* were smaller and contained less starch than the empty vector controls. This is likely because the *proUSP:TPP*-expressing embryos exhibited lower levels of expression of *AGPL*, *AGPS1*, and *AGPS2*, which encode the subunits of the starch biosynthesis enzyme AGPase. Furthermore, the *proUSP:TPP*-expressing embryos also showed reductions (up to 70%) in seed levels of 4-Cl-IAA and increases in 4-chlorotryptophan, which strongly suggests that T6P upregulates auxin biosynthesis in pea seeds. In accord with this hypothesis, *TAR2* expression was also reduced in the *proUSP:TPP*-expressing embryos, indicating a flow reduction in the IPyA pathway of auxin biosynthesis. This also explains the reduction in seed starch content, as *PsAGPL*, *PsAGPS1*, and *PSAGPS2* contain auxin-responsive elements in their promoters [45]. The additional expression of *proUSP:TAR2* alongside *proUSP:TPP* rescued seed weight and starch content, but embryos appeared less pigmented/lacking chlorophyll relative to wild type seeds [43].

Some corresponding observations were made in the *proUPS:TPS*-expressing embryos that had elevated embryonic T6P. Namely, AGPase activity was elevated relative to the wild type, as was *TAR2* expression and seed 4-Cl-IAA concentration. Seed size and starch content were unaffected in the *proUPS:TPS*-expressing embryos [43], however, implying that T6P concentration does not limit seed growth. The soluble sugar concentration was reduced in the *proUPS:TPS*-expressing embryos, implying a greater conversion of photoassimilates into T6P, but the lack of a change in absolute photoassimilate supply (relative to the wild type) likely prevented a concomitant increase in seed size and starch content. Whether multiplying grafted plants, in which a WT shoot system and *UPS:TPS*-expressing shoot system are grafted onto a common root system, would lead to alterations in the intra-plant seed size in a genotype-dependent manner remains unknown.

These brilliant results from Meitzel et al. [43] agree with previously published work which showed that mutations in *Pstar2*, a gene involved in producing chlorinated auxin from 4-Cl tryptophan, caused strong defects in seed filling and starch biosynthesis gene expression [45], implicating auxin signalling as critical in this phase of reproductive development. Furthermore, experiments in which either endogenous [46] or heterologous potato [47] sucrose transporters were overexpressed in pea phloem and/or seeds showed increases in seed sucrose and protein content [46] and faster rates of seed biomass accumulation [47].

Altogether, these results demonstrate parent-to-seed sugar signalling, mediated by the phosphorylated disaccharide trehalose 6-phosphate and its subsequent promotion of auxin biosynthesis, as a key system in the seed-filling phase of reproductive development in pea (Figure 1B). The processes underlying the transition of the pod from a flattened shape at the end of pod elongation to a rounded shape at the completion of seed filling remain to be elucidated, but the bi-functional role of 4-Cl-IAA in both pod elongation and seed filling may indicate that it is a central regulator of these developmental transitions.

## 4. Conclusions and Future Perspectives

As growing environmental pressures continue to heighten the global demand for plant protein, fundamental research into the biology of pea and other legumes is poised to play a critical role in the future of global agriculture. Pea exhibits wide intraspecific diversity in organ morphology, development, and genome sequence compared to *Arabidopsis* and other Brassicaceae. Moreover, the large and unicarpellate fruits of pea make it an alluring model for research into plant reproductive development, and the biosynthesis of its unusual 4-chlorinated auxin raises questions about the function of this novel phytohormone and how it is produced. Given the apparent centrality of 4-Cl-IAA to pea reproductive development, how other, non-4-Cl-IAA-producing legumes (e.g., *Cicer*, *Glycine*, *Phaseolus*) would respond to the introduction of the hormone by transformation remains an open and exciting question. A detailed description of the differences in reproductive signalling between crop legume species is also outstanding. We posit that research into these areas will be necessary for unlocking the crop phenotypes of the future, which can deliver in the face of unprecedented global challenges.

Specific, outstanding questions in the field of pea reproductive development include how plants obtain 4-chlorinated tryptophan as a precursor to 4-Cl-IAA, how plants are able to differentially respond to 4-Cl-IAA and IAA, whether 4-Cl-IAA and/or T6P are mobile signals between seed and pod, and whether variation in these signalling components contributes to the observed variation in pod morphology in this species and in other legumes. We anticipate that, by approaching these research questions, creative possibilities in legume agronomy will be revealed and new insights into the fundamental life process of reproduction may be gained.

## Data Availability

Not applicable.
